# Untangling the role of one-carbon metabolism in colorectal cancer risk: a comprehensive Bayesian network analysis

**DOI:** 10.1038/srep43434

**Published:** 2017-02-24

**Authors:** Robin Myte, Björn Gylling, Jenny Häggström, Jörn Schneede, Per Magne Ueland, Göran Hallmans, Ingegerd Johansson, Richard Palmqvist, Bethany Van Guelpen

**Affiliations:** 1 Department of Radiation Sciences, Oncology, Umeå University, Umeå, Sweden; 2 Department of Medical Biosciences, Pathology, Umeå University, Umeå, Sweden; 3 Department of Statistics, Umeå School of Business and Economics, Umeå University, Umeå, Sweden; 4 Department of Clinical Pharmacology, Pharmacology and Clinical Neurosciences, Umeå University, Umeå, Sweden; 5 Department of Clinical Science, University of Bergen and Laboratory of Clinical Biochemistry, Haukeland University Hospital, Bergen, Norway; 6 Department of Biobank Research, Public Health and Clinical Medicine, Umeå University, Umeå, Sweden; 7 Department of Odontology, Cariology, Umeå University, Umeå, Sweden

## Abstract

The role of one-carbon metabolism (1CM), particularly folate, in colorectal cancer (CRC) development has been extensively studied, but with inconclusive results. Given the complexity of 1CM, the conventional approach, investigating components individually, may be insufficient. We used a machine learning-based Bayesian network approach to study, simultaneously, 14 circulating one-carbon metabolites, 17 related single nucleotide polymorphisms (SNPs), and several environmental factors in relation to CRC risk in 613 cases and 1190 controls from the prospective Northern Sweden Health and Disease Study. The estimated networks corresponded largely to known biochemical relationships. Plasma concentrations of folate (direct), vitamin B6 (pyridoxal 5-phosphate) (inverse), and vitamin B2 (riboflavin) (inverse) had the strongest independent associations with CRC risk. Our study demonstrates the importance of incorporating B-vitamins in future studies of 1CM and CRC development, and the usefulness of Bayesian network learning for investigating complex biological systems in relation to disease.

One-carbon metabolism (1CM) is a metabolic network centered around the folate and methionine cycles, essential for methylation and nucleotide synthesis (Fig. 1). 1CM is vital for genome stability and function and is the target of antimetabolite/antifolate chemotherapy. An important biological role for 1CM in cancer development is therefore highly plausible^1^.

The role of 1CM in colorectal cancer (CRC) development has been extensively studied. Findings include a possible dual role for the B-vitamin folate, depending on the dose and timing of exposure (i.e., protecting healthy mucosa but promoting undiagnosed lesions^2^,^3^). For other B-vitamins, the most notable finding is an inverse association between plasma concentrations of vitamin B6 (pyridoxal 5′-phosphate, PLP) and CRC risk^4^, whereas results have been inconclusive for vitamin B2 (riboflavin) and B12 (cobalamin) status^5^. For metabolites in the transsulfuration pathway, such as homocysteine and cysteine, results have been inconclusive^6^,^7^,^8^,^9^,^10^,^11^. For metabolites primarily involved in methylation, such as methionine and factors in the choline oxidation pathway, inverse associations between colorectal adenoma and CRC risk have been observed^12^,^13^,^14^,^15^. To our knowledge, dietary intake or circulating levels of serine and glycine, important one-carbon group donors to tetrahydrofolate (THF) in the folate cycle (Fig. 1), have not been studied in relation to CRC risk, but implications in cancer cell proliferation have been observed *in vitro*^16^. Several single nucleotide polymorphisms (SNPs) in genes coding for enzymes in 1CM have also been studied^17^, with the most important finding being a reduced risk of CRC in TT genotype carriers of the methylenetetrahydrofolate reductase (*MTHFR*) 677C > T polymorphism^18^. Randomized clinical intervention trials on the effects of folic acid, vitamin B6, or vitamin B12 supplementation on colorectal adenoma or cancer occurrence have been inconclusive^19^,^20^,^21^.

The varying presence, strength, and even direction of the observed associations between compounds of 1CM and CRC risk may have several explanations, for instance, variation in levels and timing of exposure between study populations^18^. Furthermore, the commonly used method of univariate modeling of single variables, typically in multivariable models adjusting for potential confounders, may miss higher-order interactions and mediating effects. This is particularly important for 1CM, given the complexity of input (diet, supplementation, and fortification), interrelationships, gene-environment interactions, and output (nucleotide synthesis, methylation, inflammation, oxidation, and energy metabolism^22^,^23^,^24^).

To account for the complexity of 1CM in molecular epidemiology, mathematical or pathway-based modeling based on prior biochemical knowledge have been successfully applied^25^. However, no empirical study using observational data has so far addressed the complex interplay of plasma markers of 1CM, related SNPs, and environmental factors in relation to cancer risk. A Bayesian network (BN) is a graphical representation showing all independent relations among a set of variables (a network of *nodes*, representing variables, connected by lines referred to as *edges,* representing independent relations between variables). A BN can be estimated - or learned - from data using machine learning algorithms^26^. This methodology has previously been applied in studies of complex systems in several scientific disciplines^26^, including epidemiology^27^,^28^. Using BNs in studies of 1CM and cancer could provide a more comprehensive understanding of the relation between 1CM and carcinogenesis.

In this study of 613 colorectal cancer cases with prediagnostic blood samples and 1190 matched controls from the population-based Northern Sweden Health and Disease Study (NSHDS), we used Bayesian network learning to investigate, simultaneously, the relative contributions of and interplay among a comprehensive panel of 14 prediagnostic plasma one-carbon metabolites, 17 SNPs involved in 1CM, and a set of other environmental factors, in relation to CRC risk.

## Results

### Baseline characteristics

Baseline characteristics for case participants and matched control participants, and clinical characteristics for the cases, are presented in Table 1. There was a slightly larger proportion of ex-smokers and a lower proportion of never smokers among cases compared to controls. Body mass index (BMI), alcohol intake, physical activity (occupational and recreational), and B-vitamin intakes were similar for cases and controls. Vitamin supplement usage was low among both cases and controls. The median age at diagnosis was 65.2 years. Median follow-up time between blood sampling of the cases and their CRC diagnosis was 8.2 years. The tumors were roughly equally distributed by site (30% proximal, 35% distal, and 35% rectal) and stage (53% I/II and 47% III/IV).

Baseline plasma metabolite concentrations differed between cases and controls for some metabolites, but only vitamin B2 differed significantly after Bonferroni correction for multiple testing (adjusted significance threshold: 0.05/14 ≈ 0.004, Table 1). Spearman correlations between plasma concentrations of one-carbon metabolites are presented in Supplementary Fig. S1. Four groups of more highly correlated metabolites were apparent: (1) metabolites in the choline pathway (choline, betaine, DMG, and sarcosine), (2) B-vitamins (folate, vitamin B6, B2, and B12), (3) serine and glycine, and (4) methionine and metabolites in the transsulfuration pathway (methionine, homocysteine, cystathionine, and cysteine). Correlations were highest between directly related metabolites, such as choline and betaine (r = 0.40) and glycine and serine (r = 0.52). Total homocysteine was negatively correlated with both folate (r = −0.37) and vitamin B12 (r = −0.28). Methionine and cystathionine were also correlated to metabolites in the choline pathway (r = 0.17–0.28). The correlations were essentially the same for cases and controls.

Genotype distributions did not differ between cases and controls for any SNP (Supplementary Table S1). No SNPs showed significant deviations from Hardy-Weinberg equilibrium (adjusted significance threshold: 0.05/34 ≈ 0.0015, Supplementary Table S1).

### Bayesian network learning

**Figure 2:**
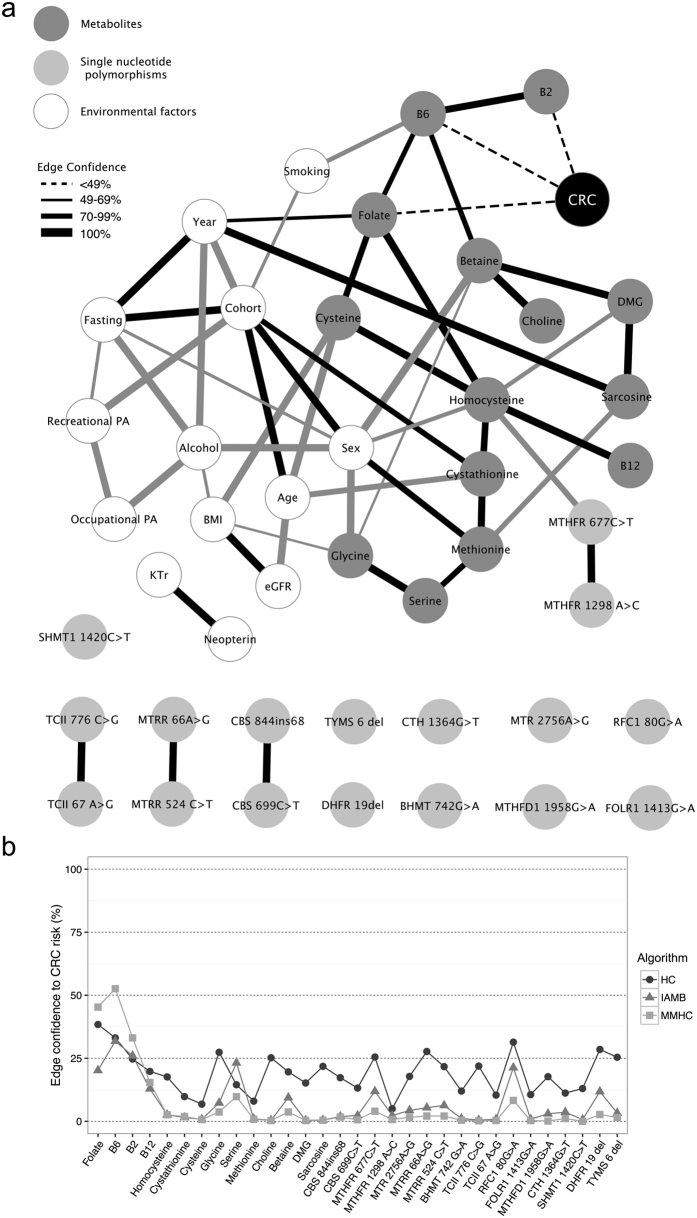
Bayesian network learning results **(a)** Bayesian network of plasma one-carbon metabolites divided into quartiles, related SNPs, and other environmental variables in relation to colorectal cancer (CRC) estimated with the HC algorithm. Analyses were made on 560 cases and 1090 controls (after excluding 53 cases and 100 controls with incomplete 1CM data). Edges in black were also present in IAMB and/or MMHC networks, whereas gray edges were present only in the HC network. Thicker edges indicate higher confidence (i.e., the frequency of the relation in the 1000 bootstrap networks). The estimated confidence thresholds for inclusion in the networks were: HC = 49%, IAMB = 50%, MMHC = 51%. The strongest independent associations with CRC risk, with edge confidences consistently higher compared to other variables for all algorithms, are marked with dashed edges. **(b)** Edge confidences of relations between CRC and 1CM variables for networks learned using the HC, IAMB, and MMHC algorithms. A higher edge confidence indicates a stronger independent association. Abbreviations: PA, physical activity; eGFR, estimated glomerular filtration rate; KTr, kynurenine/tryptophan ratio.

The combined BN estimated from data using three different algorithms, including all metabolites, SNPs, and other variables in relation to CRC, is presented in Fig. 2a. An edge (i.e., drawn line) between two variables implies an association independent of all other variables in the network. The networks estimated using the Hill-climbing (HC) algorithm had more edges compared to the networks estimated using the Incremental Association Markov Blanket (IAMB) and Min-Max Hill-climbing (MMHC) algorithms. Regarding independent associations between CRC and other variables, the overall pattern was the same for all algorithms but with slightly stronger associations for more variables in the HC networks (measured by edge confidence, i.e., the frequency of the edge in the 1000 bootstrap networks) (Fig. 2b). The edge confidence significance thresholds that needed to be met for a relation to be included in the networks were essentially the same (HC = 49%, IAMB = 49%, MMHC = 50%).

In the BNs, plasma concentrations of the metabolites were related to each other mainly according to known biochemical relationships (Fig. 2a). Homocysteine levels were related to the *MTHFR* 677C > T polymorphism. No other SNP was strongly associated with the plasma concentrations of any of the metabolites. SNPs within the same genes were associated, suggesting linkage disequilibrium. Some independent associations between environmental factors and metabolites were present. For instance, vitamin B6 was related to smoking and cysteine was related to BMI. The relation between sampling year and sarcosine (manifested as slightly higher levels in participants sampled in later years) was likely an artifact stemming from spurious amounts of sarcosine in the EDTA tubes used during that period. The BNs also picked up associations between background variables inherent to the study design (e.g., between cohort and sex, age, fasting status, and sampling year).

Folate, vitamin B6, and vitamin B2 had the strongest independent associations with CRC risk, with edge confidences consistently higher compared to other variables for all algorithms (Fig. 2b). Yet, the edge confidences were generally not above the estimated significance thresholds. The *RFC1* 80G > C polymorphism displayed a higher edge confidence to CRC compared to other SNPs, though it did not meet the threshold (Fig. 2b). Removing the metabolites from the BNs did not markedly affect the associations for SNPs.

Edge confidences for the strongest independent associations between 1CM variables and CRC risk (BNs estimated with the HC-algorithm) are presented for subgroups based on sex, follow-up, and tumor site and stage in Supplementary Table S2. In sex-specific BNs, the most apparent difference was a stronger relation between folate and CRC in men (P_heterogeneity_ = 0.004). Folate was mainly directly associated to stage III&IV cancers (P_heterogeneity_ = 0.04). Vitamin B12 was associated with rectal cancer in tumor site-specific BNs, though the test for heterogeneity was not significant (P_heterogeneity_ = 0.15). The remaining structure did not markedly differ between subgroup networks and the variables with the strongest relation to CRC or CRC subgroup were largely the same regardless of algorithm.

### Univariate and interaction analyses

**Figure 3:**
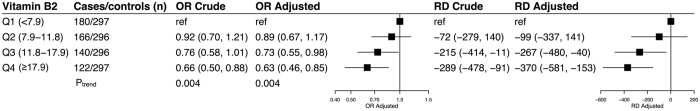
Risk of CRC by vitamin B2 status. Odds ratios (OR) were calculated by conditional logistic regression. Absolute risk differences (RD) were determined using weighted maximum likelihood estimation. Quartiles of plasma concentrations of vitamin B2 (riboflavin, nmol/l) were based on the distribution among the controls participants. Confidence intervals for the RDs were calculated by bootstrapping. Crude OR and RD estimates were adjusted only for the matching variables, using risk set stratification in conditional logistic regression and by including them as covariates in the weighted maximum likelihood models, respectively. Adjusted estimates were additionally adjusted for BMI, smoking status, occupational and recreational activity, alcohol intake, and plasma folate and vitamin B6 (PLP) concentrations. P_trend_ was calculated by modeling log-transformed plasma concentrations in conditional logistic regression models.

Plasma vitamin B2 concentrations were inversely related to CRC risk (highest vs. lowest quartile OR: 0.63, 95% CI: 0.46–0.85, P_**trend**_ = 0.004, Fig. 3). The corresponding average absolute risk reduction was approximately 300 cases per 100 000 in the highest versus lowest quartile. Adjusting for potential confounders, including folate and vitamin B6, did not markedly change the risk estimates. Univariate analyses of the other variables with the strongest relationship to CRC, i.e. folate and vitamin B6, have either been published (lower CRC risk at lower plasma folate concentrations^6^,^11^) or submitted to a scientific journal (higher CRC risk at lower plasma concentrations of vitamin B6, data not shown here).

We investigated 2-way interactions between the most influential variables: folate, vitamin B6, and vitamin B2. We observed no interaction between folate and vitamin B6 or folate and vitamin B2 (P_interaction_ = 0.29 and 0.16, respectively), whereas vitamin B2 and B6 exhibited a significant interaction (P_interaction_ = 0.004). Table 2 contains ORs for CRC risk by combinations of vitamins B2 and B6 levels estimated with the fitted parameters of the BN and conditional logistic regression including interaction terms (with plasma concentrations divided in tertiles to avoid spurious associations). The inverse association between vitamin B2 and CRC risk was attenuated at higher levels of vitamin B6, with the highest risk observed in the low-low category. ORs calculated from the BN were approximately the same as ORs from conditional logistic regression models.

### Sensitivity analyses

Since categorization of plasma metabolites may result in loss of information, we estimated BNs using finer categorization (septiles, representing a balance between increasing the number of categories and maintaining adequate numbers in each category). This analysis did not markedly change the resulting networks, with the exception of a moderate increase in confidence for the independent association between vitamin B2 and CRC.

Since undiagnosed cancer may affect plasma metabolite levels at the time of sampling (reverse causation), we estimated BNs excluding cases diagnosed within 1 years (25 cases) or 2 years (60 cases) of sampling, and their corresponding matched controls. This analysis did not markedly change the resulting networks.

As plasma metabolite concentrations can vary by fasting status, we estimated BNs excluding participants with fasting status less than 4 hours (141 cases and 272 controls). This analysis did not markedly change the resulting networks. In univariate analyses, the associations for folate, vitamin B6, and vitamin B2 did not differ by fasting status (P_heterogeneity_ = 0.35, 0.80, and 0.28, respectively).

## Discussion

In this population-based case-control study, a comprehensive panel of metabolites and SNPs involved in 1CM along with several environmental factors were analyzed simultaneously by Bayesian network learning to study interrelations and relative contributions to CRC risk. The associations represented in the estimated networks largely corresponded to plausible biochemical relationships. Plasma concentration of folate, vitamin B6 (PLP), and vitamin B2 (riboflavin) had the strongest independent relations to CRC risk. In multivariable, univariate analyses, vitamin B2 demonstrated a linear inverse association with CRC risk. Vitamin B6 significantly modified this relation.

This is the first time Bayesian network learning, or any similar multivariate statistical approach, has been applied to investigate 1CM in relation to any cancer. Bayesian network learning allows many variables to be modeled simultaneously to study all relations in a system. In our study, the estimated BNs identified known and biologically plausible associations between factors, which underscores the validity of the method. Bayesian network learning does not replace traditional methods but is a valuable exploratory tool for understanding independent associations among multiple variables and to facilitate proper selection of variables to consider in further univariate analyses. This is particularly relevant in studies of biological systems with many highly interrelated environmental and genetic factors, such as 1CM.

The independent associations between prediagnostic plasma concentrations of folate, vitamin B6, and vitamin B2 and CRC risk suggest that they may be the 1CM components of greatest importance in colorectal tumorigenesis. Low plasma folate concentrations were associated with a decreased CRC risk (previously published^6^,^11^), whereas low plasma concentrations of vitamins B6 and B2 were associated with an increased CRC risk. These observations are consistent with previous findings from univariate modeling, both in our data^6^,^11^ and other studies^5^. The relative importance and interconnectedness of B-vitamins in cancer development are also consistent with results from animal studies and mathematical modeling of 1CM^29^. Folate and vitamins B2 and B6 are involved in DNA synthesis and methylation, biological processes important for genome stability and repair^1^. Since these functions are critical in both the healthy colorectum and in tumorous lesions, a cancer-promoting effect has been proposed for folate^2^,^3^, consistent with the direct association with CRC risk observed in this study. As cofactors in the kynurenine pathway^23^,^24^,^30^, both B6 and B2 are linked to inflammation, a process known to influence cancer development^31^, though biomarkers of systemic inflammation available in this study (plasma neopterin and the kynurenine/tryptophan ratio) were not strongly associated with either B-vitamin, nor did they alter the relation between the B-vitamins and CRC risk. Vitamin B6 and B2 are also cofactors in a large number of other coenzyme reactions in macronutrient metabolism^5^, and vitamin B6 has been suggested to reduce oxidative stress, colon cancer cell proliferation, and angiogenesis^32^. The inverse associations between vitamin B6 and B2 and CRC risk in this study may, therefore, reflect other mechanisms than 1CM.

There were some differences in the most influential variables when we estimated BNs on stratified data. Independent associations between plasma folate concentrations and CRC were observed in men and for stage III and IV CRC, which is not entirely consistent with our previous findings based on multivariable, univariate modeling^6^,^11^. The independent association between plasma vitamin B12 concentrations and rectal cancer risk supports our previous findings^6^,^11^,^33^. Structural learning algorithms are less efficient in smaller sample sizes, especially in networks with many interrelations^34^. The results of the subgroup analyses must, therefore, be verified in larger data sets.

Among the examined SNPs, the only independent association observed was between the *MTHFR* 677C > T polymorphism and plasma homocysteine levels. Interestingly, none of the polymorphisms exhibited a strong independent relation to CRC risk. In previous univariate analyses of the same data, we found a small CRC risk reduction in individuals with the variant CT or TT genotype of the *MTHFR* 677C > T polymorphism^11^,^35^. The largely null findings for the SNPs in this study might, therefore, reflect a mediating effect through altered metabolite levels. This would be consistent with the premise of Mendelian randomization studies, for which MTHFR 677C > T is a commonly studied example. However, our results were not markedly affected by removing the plasma metabolites from the BNs.

The main limitation of this study was the analysis of only one blood sample from each participant. On the other hand, issues of storage stability and reproducibility of the included biomarkers are well studied and unlikely to have impacted the results markedly^36^,^37^. Although common practice, categorizing continuous variables (e.g., dividing plasma concentrations into quartiles) results in loss of information^26^. However, in a sensitivity analysis, BNs estimated using septile categories yielded similar results. Alcohol intake and physical activity were only available for the VIP cohort, which may have caused residual confounding. The majority of the data were from VIP (78%), and we included several other related environmental factors (e.g., BMI, smoking status, and inflammatory markers). A large impact of residual confounding on the main findings is, therefore, unlikely. Last, we were not able to validate the estimated network in an independent data set. However, we evaluated the robustness of our associations by a bootstrapping approach, and both known biochemical relationships and associations between background variables inherent to the study design were largely picked up by the Bayesian networks. Furthermore, the variables with the strongest independent relations to CRC risk in the networks (folate, B6, and B2), demonstrated associations and interrelationships consistent with previous reports, and were also significant in traditional univariate logistic regression models. Taken together, these observations support the validity of our findings.

The main strength of our study was the application, for the first time in a study of cancer risk, of multivariate statistical methods to a large panel of well-characterized circulating one-carbon metabolites, SNPs, and environmental factors. We tested several structural learning algorithms, yielding similar results regarding the strongest relations to CRC risk. In the overall network structure, networks estimated with the HC algorithm resulted in more edges than the IAMB and MMHC algorithms. This is likely explained by the higher sensitivity and better overall performance of the HC algorithm compared to the IAMB and MMHC algorithms, previously demonstrated in simulations^34^. Another strength of the study was the use of prediagnostic blood samples of high quality with respect to the collection, handling, and storage, including a majority of fasting participants. Follow-up time from sampling to CRC diagnosis was long (median 8.2 years), which minimized the risk of reverse causation. Furthermore, the study population from northern Sweden is generally characterized by low folate levels^38^,^39^,^40^. This allowed us to study the effects of much lower plasma folate concentrations in relation to CRC risk compared to other studies^40^,^41^.

In conclusion, this is the first study to address the complexity of 1CM in cancer risk in humans. We used multivariate Bayesian network learning to estimate, simultaneously, the associations of a comprehensive panel of prediagnostic plasma metabolites and SNPs involved 1CM and the risk of CRC. The associations between components of 1CM and CRC risk were mainly determined by variation in folate, vitamin B6, and vitamin B2 status, suggesting that these may be the elements of 1CM with the greatest potential impact for CRC prevention strategies. Our study demonstrates the importance of incorporating these B-vitamins in future studies of 1CM in colorectal cancer development, and the usefulness of Bayesian network learning in studies of complex biological systems in relation to disease.

## Methods

### Study design and cohorts

The present work is based on a nested case-control study within the Northern Sweden Health and Disease Study (NSHDS). Two population-based cohorts were used, the Västerbotten Intervention Programme (VIP, 78% of the study participants, men and women) and the Mammography Screening Project in Västerbotten (MSP, 22% of the study participants, all women). Both cohorts have previously been described in detail^42^. As of March 31, 2009, the final date for case identification for the present study, the VIP included 83 621 individuals and 114 793 blood samples, and the MSP 28 802 women and 54 787 blood samples. Selection bias in the VIP has been found to be low^43^, and the population-based nature of the VIP cohort is supported by comparisons of cancer incidence rates^44^.

### Study participants

CRC cases diagnosed between October 17, 1986, and March 31, 2009, who had donated prediagnostic blood samples, were identified by linkage with the Cancer Registry of Northern Sweden (ICD-10 18.0 and 18.2–18.9 for colon, 19.9 and 20.9 for rectum), with essentially complete inclusion. All cases, as well as tumor data, were verified by a single pathologist specialized in gastrointestinal pathology. Patient records were used to verify tumor site. Exclusion criteria included: previous cancer diagnosis other than non-melanoma skin cancer, insufficient volume of plasma sample available, prioritizing to other studies, location of primary tumor outside the colon/rectum, serious infectious diseases (for lab staff safety, one case excluded), or no matching control obtainable.

Two controls were randomly selected for each case, matched by sex, age at and year of blood sampling and data collection, fasting status, and cohort. The exclusion criteria for the controls were the same as for cases, with the additional requirement that all controls had to be alive and with no diagnosed cancer other than non-melanoma skin cancer at the time of diagnosis of their index cases.

A total of 613 cases and 1190 controls were in included in the study after exclusions. In total, 127 participants were excluded (81 cases and 46 controls), mainly due to insufficient blood sample volume or to the sample being prioritized to other studies. A detailed description of the exclusions is available elsewhere^35^.

The subjects in the present study have previously been separately analyzed for eight of the plasma metabolites (folate, cobalamin, homocysteine, methionine, choline, betaine, dimethylglycine, and sarcosine), four of the polymorphisms (*MTHFR* 677C > T and 1298A > C*, BHMT* 742G > A, and *MTR* 2756A > G)^6^,^11^,^33^,^35^, and for subjects with index case diagnosis 1986–2003, also the *RFC1* 80G > A and *FOLR1* 1413G > A polymorphisms^35^,^45^, in relation to colorectal cancer (CRC) risk. A total of 17 CRC cases and 33 controls in the present study were also included in previous studies within the European Prospective Investigation into nutrition and Cancer (EPIC)^14^,^46^.

### Ethical considerations

The study protocol was approved by the Research Ethics Committee of Umeå University, Umeå, Sweden. All participants gave a written informed consent. All analyses were conducted in accordance with relevant guidelines and regulations.

### Blood sampling and laboratory analyses

Plasma from venous blood samples in the NSDHS is aliquoted and cryopreserved at −80 °C within one hour of collection, or at −20 °C for at most one week prior to long-term storage at −80 °C. In the VIP cohort, samples are collected in the morning, and only 34 of 1410 participants (2%) had fasted less than 4 hours and 295 (21%) less than 8 hours. In the MSP cohort, samples were collected throughout the day, and 379 of 393 participants (96%) had fasted less than 4 hours. Thus, in the total material, 60% of the participants had fasted for more than 8 hours, 17% had fasted 4–8 hours, and 23% had fasted less than 4 hours. Concentrations of 1CM metabolites in EDTA plasma and polymorphisms involved in 1CM were analyzed at Bevital AS (Bergen, Norway)^47^. Plasma concentrations of cystathionine, vitamin B2 (riboflavin), vitamin B6 (PLP), methionine, choline, betaine, dimethylglycine, creatinine, neopterin, and tryptophan were measured with liquid chromatography–mass spectrometry methods (between-day coefficient of variation (CV): 3–13%)^48^. Plasma concentrations of total homocysteine, total cysteine, serine, glycine, sarcosine, and kynurenine were measured using an isotope dilution gas chromatography–mass spectrometry method (between-day CV: 2–9%)^49^. Folate and vitamin B12 (cobalamin) concentrations were determined with a microbiological method using *Lactobacillus casei* and *Lactobacillus leichmannii*, respectively, which was adapted to a microtiter plate format and carried out by a robotic workstation (between-day CV: 5%)^50^,^51^. Single nucleotide polymorphisms were determined using MALDI-TOF mass spectrometry (estimated average error rate of ≤0.1% in duplicated samples)^52^. The genotyping method has previously been independently verified using RFLP or Taqman real-time PCR^52^. Samples were analyzed in case-control sets, with random positioning of the case. The investigators and laboratory staff were blinded to case and control status.

### Variables

Plasma concentrations of 14 metabolites and 17 SNPs in 13 genes involved in, or related to, 1CM were considered for the Bayesian network learning. The panel was designed based on previous studies of 1CM and CRC risk^5^,^17^, and to capture a wide array of aspects of one-carbon metabolism while maintaining an adequate marker stability and reproducibility^36^,^37^. Included metabolites were: folate, vitamin B6 (PLP), vitamin B2 (riboflavin), vitamin B12 (cobalamin), homocysteine, cystathionine, cysteine, glycine, serine, methionine, choline, betaine, dimethylglycine, and sarcosine. Included SNPs were: *MTHFR* 677C > T and 1298A > C, *CBS* 844ins68 and 699C > T, *MTR* 2756A > G, *MTRR* 66A > G and 524C > T, *BHMT* 742G > A, *TCN2* 67A > G and 776C > G, *RFC1* 80G > A, *FOLR1* 1413G > A, *MTHFD1* 1958G > A, *CTH* 1364G > T, *SHMT1* 1420C > T, *DHFR* 19 deletion, and *TYMS* 6 deletion. Other environmental factors or background information included were: cohort (VIP or MSP), age at and year of blood sampling (quartiles), sex (male or female), fasting status (<4, 4–8, ≥8 hours), smoking status (current, ex-, never smoker), body mass index (BMI) measured by a health professional (<25, 25–30, ≥30 kg/m^2^), estimated glomerular filtration rate (eGFR) calculated by the Cockcroft-Gault formula (based on plasma creatinine levels, age, sex and body weight, quartiles), and plasma concentrations of neopterin and the kynurenine/tryptophan ratio (KTr), both markers of immune activation^53^,^54^ (quartiles). For the VIP cohort, we also had self-reported alcohol intake (zero intake, above/below sex-specific median of self-reported, g/day), recreational physical activity (regular exercise frequency on a scale from 1–5, where 1: never; 2: every now and then - not regularly; 3: 1–2 times/week; 4: 2–3 times/week; 5: more than 3 times/week), and occupational physical activity (on a scale from 1–5, where 1: sedentary or standing work; 2: light but partly physically active; 3: light and physically active; 4: sometimes physically strenuous; 5: physically strenuous most of the time). For these VIP-only variables, observations within the MSP cohort were assigned to a separate “missing” category.

Plasma concentration variables were analyzed in quartile groups (cut-offs based on the distribution of the controls). The *CBS* 844ins68, *TCN2* 67A > G, and *FOLR1* 1413G > A SNPs were analyzed in two groups, common and variant genotype, because of low allele frequencies (4, 36, and 3 individuals with the homozygous variant genotype respectively). All other SNPs were analyzed in three categories: common, heterozygous, and homozygous variant genotype.

Missing values for plasma metabolites and SNPs were assumed to be missing completely at random and were therefore omitted from the analyses (0–3% missing per variable). Missing values for the environmental factors were assigned to separate categories. Thus, the Bayesian network learning was conducted on 560 cases and 1090 controls with complete 1CM data.

### Statistical analyses

All computations were conducted in R v.3.2.4^55^. Network visualizations were created using Cytoscape v.3.2.1^56^. All statistical tests were two-sided with a significant threshold of 0.05.

Mann-Whitney U test or Chi-square tests were used to test for differences in variable distributions between cases and controls. Correlations between plasma metabolite variables in all subjects were calculated with Spearman’s correlation coefficient on pairwise complete observations. A hierarchical cluster analysis of the metabolites was conducted using correlation distances with complete linkage. Pearson’s χ^2^-test was used to check if SNPs were in Hardy-Weinberg equilibrium for cases and controls separately when the expected cell count was above 5, otherwise Fisher’s exact test was used. The significance thresholds for the tests were corrected for multiple testing with the Bonferroni method.

The BNs were estimated on discrete data with a model-averaging approach based on bootstrapping^34^. In 1000 bootstrap samples, BNs were estimated with three different machine learning algorithms using the *boot.strength* function in the bnlearn R-package. Then, the final networks were obtained by averaging over the 1000 bootstrap networks using the *averaged.network* function. An edge was included if its edge confidence, defined as the frequency of occurrence of that relation among the 1000 bootstrap networks, was above a threshold based on observed confidence levels^34^. The three machine learning algorithms used in each bootstrap sample were the score-based Hill-climbing (HC), the constraint-based Incremental Association Markov Blanket (IAMB), and the hybrid Min-Max Hill-climbing (MMHC) algorithms. The scoring function for the HC and MMHC algorithms was the Akaike information score (AIC) and the conditional independence test for the IAMB and MMHC algorithms was the asymptotic χ^2^ mutual information test.

Univariate risk estimates in the form of odds ratios (ORs) for the 1CM variables with a pronounced relation to CRC in the BNs, and for which we have not previously published results or submitted results to a scientific journal, were computed with conditional logistic regression. Linear trends for the metabolites were tested by modeling log-transformed plasma concentrations. Absolute risk estimates, defined as marginal risk differences (RDs), were computed with a weighted maximum likelihood estimator using cumulative incidence data from the study cohort at large, and within groups defined by sampling year, age, sex, and cohort (cumulative incidence of CRC in the study cohort was 830 per 100 000 over the period 1987–2009)^35^. We present both risk estimates from unadjusted models and from models adjusted for potential confounders. Adjusted estimates were adjusted for BMI, smoking status, occupational and recreational activity, alcohol intake, and plasma B-vitamins folate and vitamin B6 (PLP) concentrations. We evaluated 2-way interactions between variables demonstrating the strongest independent relations to CRC risk in the BNs. Conditional probabilities calculated from estimated parameters of the networks were used to determine ORs over combinations of the variables with the *cpquery* function in the *bnlearn* R-package. ORs from conditional logistic regression models fitted with interaction terms were also calculated. The overall significance of the interactions was evaluated by fitting interaction terms using log-transformed metabolite concentrations or treating SNPs as continuous variables (labeled 0,1 and 2, representing copies of the less common allele).

Heterogeneity of the associations was evaluated by estimating BNs on data stratified by sex, follow-up time from blood sampling to diagnosis (above or below median follow-up of 8.2 years), tumor site (proximal colon, distal colon or rectum), and tumor stage (I&II or III&IV). For variables that appeared to differ among the stratified BNs, we further evaluated heterogeneity with likelihood ratio tests using conditional logistic regression. The likelihood ratio tests used compared a conditional logistic regression model in which the risk association could vary across endpoints to a model in which all associations were held constant (or for interactions with sex: comparing a model with product terms to a model without)^57^.

## Additional Information

**How to cite this article:** Myte, R. *et al*. Untangling the role of one-carbon metabolism in colorectal cancer risk: a comprehensive Bayesian network analysis. *Sci. Rep.* **7**, 43434; doi: 10.1038/srep43434 (2017).

**Publisher's note:** Springer Nature remains neutral with regard to jurisdictional claims in published maps and institutional affiliations.

## Supplementary Material

Supplementary Materials

## Figures and Tables

**Figure 1:**
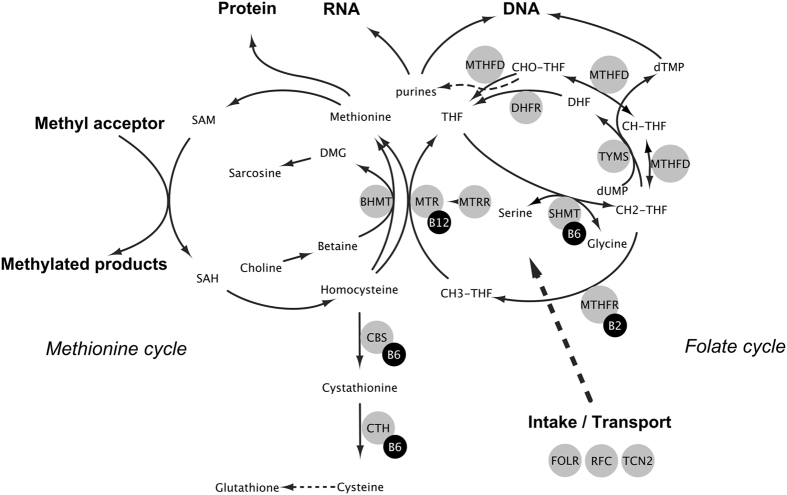
One-carbon metabolism. Graphical representation of the main aspects of one-carbon metabolism centered around the folate and methionine cycles. Abbreviations: BHMT, betaine homocysteine S- methyltransferase; CBS, cystathionine β-synthase; CH2THF, 5,10-methylenetetrahydrofolate; CH3THF, 5-methyltetrahydrofolate; CHOTHF, formyltetrahydrofolate; CHTHF, methenyltetrahydrofolate; CTH, cystathionine γ-lyase (also abbreviated CSE); DHF, dihydrofolate; DHFR, dihydrofolate reductase; dTMP, deoxythymidine 5′-monophosphate; dUMP, deoxyuridine 5′-monophosphate; FOLR, folate receptor; MTHFD, methylenetetrahydrofolate dehydrogenase; MTHFR, 5,10-methylenetetrahydrofolate reductase; MTR, methionine synthase; MTRR, methionine synthase reductase; RFC, reduced folate carrier; SAM, S-adenosylmethionine; SAH, S-adenosylhomocysteine; SHMT, serine hydroxymethyltransferase; TCN2, Transcobalamin II; THF, tetrahydrofolate; TYMS, thymidylate synthase.

**Table 1:** Baseline characteristics.

	Cases (n = 613)		Controls (n = 1190)		P^b^	Missing (%)	
	N	Median (IQR^a^) or %	N	Median (IQR^a^) or %			
**Participant characteristics**							
Age at sampling (years)	613	59.8 (40.1–67.8)	1190	59.7 (40.0–67.8)		0	
Sex, female	360	59%	703	59%		0	
Cohort, VIP	479	78%	931	78%		0	
Fasting status						0	
<4 hours	141	23%	272	23%			
4–8 hours	107	17%	202	17%			
≥8 hours	365	60%	716	60%			
Smoking status						0	
Current	114	19%	239	20%	0.01		
Ex-	136	22%	197	17%			
Never	363	59%	754	63%			
Body mass index (BMI, kg/m^2^)	591	25.7 (23.5–28.2)	1147	25.6 (23.3–28.1)	0.43	4	
Alcohol intake (g/day)^c^	385	2.4 (0.2–5.8)	754	2.3 (0.3–5.7)	0.94	13^c^	
Recreational physical activity^c^,^d^						3^c^	
1	221	45%	354	40%	0.92		
2	117	25%	240	27%			
3	81	17%	168	19%			
4	29	6%	68	7%			
5	30	7%	64	7%			
Occupational physical activity^c^,^e^					0.94	13^c^	
1	90	21%	163	20%			
2	77	18%	155	19%			
3	111	27%	223	27%			
4	102	25%	229	28%			
5	36	9%	45	6%			
eGFR (ml/min/1.73 m^2^)	596	62.0 (52.3–73.0)	1149	61.7 (50.9–72.3)	0.48	4	
Neopterin (nmol/L)	608	9.7 (8.2–11.9)	1186	9.4 (8.0–11.4)	0.02	0.5	
KTr	613	0.023 (0.019–0.026)	1190	0.022 (0.019–0.026)	0.23	0	
Vitamin supplement use^c^						0^c^	
Last 14 days	32	7%	73	8%	0.49		
Last year	40	8%	105	11%	0.11		
**Dietary B-vitamin intakes**^c^,^f^							
Folate (μg/MJ)	359	29.9 (25.3–36.0)	697	29.8 (25.3–36.0)	0.81	6^c^	
Vitamin B6 (mg/MJ)	359	0.27 (0.22–0.31)	697	0.26 (0.23–0.30)	0.98	6^c^	
Vitamin B2 (riboflavin, mg/MJ)	359	0.19 (0.16–0.22)	697	0.19 (0.16–0.22)	0.38	6^c^	
Vitamin B12 (cobalamin, μg/MJ)	359	0.61 (0.46–0.78)	697	0.59 (0.48–0.78)	0.89	6^c^	
**Plasma metabolites**							
Folate (nmol/L)	613	7.3 (4.9–10.4)	1190	7.2 (4.6–10.2)	0.49	0	
Vitamin B6 (PLP, nmol/L)	608	35.9 (25.9–51.5)	1186	38.2 (28.0–51.5)	0.08	0.5	
Vitamin B2 (riboflavin, nmol/L)	608	10.8 (7.4–16.0)	1186	11.8 (7.9–17.9)	0.002	0.5	
Vitamin B12 (cobalamin, nmol/L)	603	413 (337–498)	1173	426 (353–501)	0.02	2	
Homocysteine (μmol/L)	613	10.1 (8.4–11.9)	1190	9.9 (8.2–11.7)	0.15	0	
Cystathionine (μmol/L)	613	0.15 (0.12–0.21)	1190	0.16 (0.12–0.22)	0.65	0	
Cysteine (μmol/L)	613	275 (253–299)	1190	276 (255–298)	0.74	0	
Glycine (μmol/L)	613	227 (194–265)	1190	225 (194–277)	0.96	0	
Serine (μmol/L)	613	108 (96–122)	1190	110 (96–124)	0.43	0	
Methionine (μmol/L)	613	25.9 (23.2–29.1)	1190	26.4 (23.4–29.9)	0.07	0	
Choline (μmol/L)	606	8.6 (7.6–9.7)	1189	8.6 (7.6–9.8)	0.83	0.4	
Betaine (μmol/L)	606	29.9 (25.7–34.4)	1189	30.8 (26.1–35.5)	0.05	0.4	
DMG (μmol/L)	606	3.6 (2.9–4.4)	1189	3.6 (3.0–4.4)	0.79	0.4	
Sarcosine (μmol/L)	613	1.5 (1.1–2.0)	1190	1.5 (1.2–2.1)	0.54	0	
**Case characteristics**							
Age at diagnosis (years)	613	65.2 (59.3–70.2)				0	
Follow-up time (years)	613	8.2 (4.7–11.9)				0	
Tumor site						0.2	
Right colon	183	30%					
Left colon	215	35%					
Rectum	214	35%					
Tumor stage						5	
I-II	308	53%					
III-IV	276	47%					

Abbreviations: PLP, pyridoxal 5′ phosphate – DMG, Dimethylglycine – MJ, Mega Joules – eGFR, estimated glomerular filtration rate (by Cockcroft–Gault formula) – KTr, kynurenine/tryptophan ratio.

^a^IQR: Interquartile range (25th-75th percentile).

^b^From test for difference in distribution between cases and controls. Mann-Whitney U tests for continuous variables, Chi-square tests for categorical variables. Not calculated for matching variables. Bonferroni-corrected threshold for significance differences among metabolites = 0.05/14 ≈ 0.004.

^c^Variables available in the VIP cohort only.

^d^Self-reported exercise frequency during leisure time on a scale from 1–5, where 1: never; 2: every now and then - not regularly; 3: 1–2 times/week; 4: 2–3 times/week; 5: more than 3 times/week.

^e^Self-reported on a scale from 1–5, where 1: sedentary or standing work; 2: light but partly physically active; 3: light and physically active; 4: sometimes physically strenuous; 5: physically strenuous most of the time.

^f^Estimated from self-administered, semi-quantitative food frequency questionnaires (FFQs) designed to measure intakes during the previous year in mass/day, divided by total energy intake.

**Table 2:** Risk of CRC by vitamin B2 and B6 status.

Vitamin B2^a^	Vitamin B6^a^		
	Tertile 1 (<30.8)	Tertile 2 (30.8–45.6)	Tertile 3 (≥45.6)
Tertile 1 (<9.0)			
Cases/controls (n)	105/148	76/151	46/97
OR-BN^b^	ref	0.78	0.80
OR-CLR (95% CI)^c^	ref	0.70 (0.48, 1.02)	0.67 (0.44, 1.04)
Tertile 2 (9.0–15.3)			
Cases/controls (n)	78/138	64/134	73/123
OR-BN^b^	0.77	0.69	0.79
OR-CLR (95% CI)^c^	0.78 (0.54, 1.14)	0.66 (0.44, 0.99)	0.83 (0.56, 1.21)
Tertile 3 (≥15.3)			
Cases/controls (n)	38/110	50/110	78/175
OR-BN^b^	0.53	0.70	0.61
OR-CLR (95% CI)^c^	0.48 (0.31, 0.75)	0.63 (0.41, 0.97)	0.62 (0.43, 0.90)
P_interaction_^d^ = 0.004			

OR: Odds ratio - CI: Confidence interval - BN: Bayesian Network - CLR: conditional logistic regression – PLP: Pyridoxal 5′ phosphate.

^a^Concentrations in nmol/l, cut-offs based on the distribution of the controls.

^b^Estimated using fitted parameters of the estimated BN, additionally adjusted for the matching variables.

^c^Estimates from a CLR-model adjusted for the matching variables by risk set stratification. Adjusting for other potential confounders had essentially no effect on the estimates.

^d^Calculated by modeling log-transformed variables as multiplicative interaction terms in a CLR-model.

## References

[b1] Locasale J. W. Serine, glycine and one-carbon units: cancer metabolism in full circle. Nat Rev Cancer 13, 572–583, doi: 10.1038/nrc3557 (2013). 23822983 PMC3806315

[b2] Kim Y. I. Folate and colorectal cancer: an evidence-based critical review. Mol Nutr Food Res 51, 267–292, doi: 10.1002/mnfr.200600191 (2007). 17295418

[b3] Ulrich C. M. & Potter J. D. Folate and cancer–timing is everything. JAMA 297, 2408–2409, doi: 10.1001/jama.297.21.2408 (2007). 17551134

[b4] Zhang X. H., Ma J., Smith-Warner S. A., Lee J. E. & Giovannucci E. Vitamin B6 and colorectal cancer: current evidence and future directions. World J Gastroenterol 19, 1005–1010, doi: 10.3748/wjg.v19.i7.1005 (2013). 23467420 PMC3581987

[b5] Song M., Garrett W. S. & Chan A. T. Nutrients, foods, and colorectal cancer prevention. Gastroenterology 148, 1244–1260 e1216, doi: 10.1053/j.gastro.2014.12.035 (2015). PMC4409470 25575572

[b6] Gylling B. . Low folate levels are associated with reduced risk of colorectal cancer in a population with low folate status. Cancer Epidemiol Biomarkers Prev 23, 2136–2144, doi: 10.1158/1055-9965.EPI-13-1352 (2014). 25063522

[b7] Miller J. W. . Homocysteine, cysteine, and risk of incident colorectal cancer in the Women’s Health Initiative observational cohort. Am J Clin Nutr 97, 827–834, doi: 10.3945/ajcn.112.049932 (2013). 23426034 PMC3607656

[b8] Le Marchand L. . Plasma levels of B vitamins and colorectal cancer risk: the multiethnic cohort study. Cancer Epidemiol Biomarkers Prev 18, 2195–2201, doi: 10.1158/1055-9965.EPI-09-0141 (2009). 19661077 PMC2978659

[b9] Lee J. E. . Prospective study of plasma vitamin B6 and risk of colorectal cancer in men. Cancer Epidemiol Biomarkers Prev 18, 1197–1202, doi: 10.1158/1055-9965.EPI-08-1001 (2009). 19336555 PMC2755048

[b10] Weinstein S. J. . One-carbon metabolism biomarkers and risk of colon and rectal cancers. Cancer Epidemiol Biomarkers Prev 17, 3233–3240, doi: 10.1158/1055-9965.EPI-08-0459 (2008). 18990766 PMC2656360

[b11] Van Guelpen B. . Low folate levels may protect against colorectal cancer. Gut 55, 1461–1466, doi: 10.1136/gut.2005.085480 (2006). 16638790 PMC1856405

[b12] Bae S. . Plasma choline metabolites and colorectal cancer risk in the Women’s Health Initiative Observational Study. Cancer Res 74, 7442–7452, doi: 10.1158/0008-5472.CAN-14-1835 (2014). 25336191 PMC4268282

[b13] de Vogel S. . Biomarkers related to one-carbon metabolism as potential risk factors for distal colorectal adenomas. Cancer Epidemiol Biomarkers Prev 20, 1726–1735, doi: 10.1158/1055-9965.EPI-11-0359 (2011). 21693628

[b14] Nitter M. . Plasma methionine, choline, betaine, and dimethylglycine in relation to colorectal cancer risk in the European Prospective Investigation into Cancer and Nutrition (EPIC). Ann Oncol 25, 1609–1615, doi: 10.1093/annonc/mdu185 (2014). 24827130

[b15] Shrubsole M. J. . Associations between S-adenosylmethionine, S-adenosylhomocysteine, and colorectal adenoma risk are modified by sex. Am J Cancer Res 5, 458–465 (2015). 25628954 PMC4300688

[b16] Labuschagne C. F., van den Broek N. J., Mackay G. M., Vousden K. H. & Maddocks O. D. Serine, but not glycine, supports one-carbon metabolism and proliferation of cancer cells. Cell Rep 7, 1248–1258, doi: 10.1016/j.celrep.2014.04.045 (2014). 24813884

[b17] Figueiredo J. C., Levine A. J., Crott J. W., Baurley J. & Haile R. W. Folate-genetics and colorectal neoplasia: what we know and need to know next. Mol Nutr Food Res 57, 607–627, doi: 10.1002/mnfr.201200278 (2013). 23401104

[b18] Kennedy D. A. . Folate Intake, MTHFR Polymorphisms, and the Risk of Colorectal Cancer: A Systematic Review and Meta-Analysis. J Cancer Epidemiol 2012, 952508, doi: 10.1155/2012/952508 (2012). 23125859 PMC3483802

[b19] Ebbing M. . Cancer incidence and mortality after treatment with folic acid and vitamin B12. JAMA 302, 2119–2126, doi: 10.1001/jama.2009.1622 (2009). 19920236

[b20] Vollset S. E. . Effects of folic acid supplementation on overall and site-specific cancer incidence during the randomised trials: meta-analyses of data on 50,000 individuals. Lancet 381, 1029–1036, doi: 10.1016/S0140-6736(12)62001-7 (2013). 23352552 PMC3836669

[b21] Cole B. F. . Folic acid for the prevention of colorectal adenomas: a randomized clinical trial. JAMA 297, 2351–2359, doi: 10.1001/jama.297.21.2351 (2007). 17551129

[b22] Ueland P. M. Choline and betaine in health and disease. J Inherit Metab Dis 34, 3–15, doi: 10.1007/s10545-010-9088-4 (2011). 20446114

[b23] Ulvik A. . Evidence for increased catabolism of vitamin B-6 during systemic inflammation. Am J Clin Nutr 100, 250–255, doi: 10.3945/ajcn.114.083196 (2014). 24808485

[b24] Abbenhardt C. . Biomarkers of one-carbon metabolism are associated with biomarkers of inflammation in women. J Nutr 144, 714–721, doi: 10.3945/jn.113.183970 (2014). 24647390 PMC3985828

[b25] Thomas D. C. . Use of pathway information in molecular epidemiology. Hum genomics 4, 21 (2009). 21072972 10.1186/1479-7364-4-1-21 PMC2999471

[b26] Daly R., Shen Q. & Aitken S. Learning Bayesian networks: approaches and issues. Knowl Eng Rev 26, 99–157, doi: 10.1017/S0269888910000251 (2011).

[b27] Rodin A., Mosley T. H., Clark A. G., Sing C. F. & Boerwinkle E. Mining genetic epidemiology data with Bayesian networks application to APOE gene variation and plasma lipid levels (vol 12, pg 1, 2005). Bioinformatics 22, 1–1, doi: 10.1093/bioinformatics/bti813 (2006). PMC1201451 15725730

[b28] Su C., Andrew A., Karagas M. R. & Borsuk M. E. Using Bayesian networks to discover relations between genes, environment, and disease. BioData Min 6, 6, doi: 10.1186/1756-0381-6-6 (2013). 23514120 PMC3614442

[b29] Mason J. B. Unraveling the complex relationship between folate and cancer risk. BioFactors 37, 253–260, doi: 10.1002/biof.174 (2011). 21915934

[b30] Theofylaktopoulou D. . Vitamins B2 and B6 as determinants of kynurenines and related markers of interferon-gamma-mediated immune activation in the community-based Hordaland Health Study. Br J Nutr 112, 1065–1072, doi: 10.1017/S0007114514001858 (2014). 25105221

[b31] Grivennikov S. I., Greten F. R. & Karin M. Immunity, inflammation, and cancer. Cell 140, 883–899, doi: 10.1016/j.cell.2010.01.025 (2010). 20303878 PMC2866629

[b32] Matsubara K., Komatsu S., Oka T. & Kato N. Vitamin B6-mediated suppression of colon tumorigenesis, cell proliferation, and angiogenesis (review). J Nutr Biochem 14, 246–250 (2003). 12832027 10.1016/s0955-2863(03)00009-3

[b33] Dahlin A. M. . Plasma vitamin B12 concentrations and the risk of colorectal cancer: a nested case-referent study. Int J Cancer 122, 2057–2061, doi: 10.1002/ijc.23299 (2008). 18092327

[b34] Scutari M. & Nagarajan R. Identifying significant edges in graphical models of molecular networks. Artif Intell Med 57, 207–217, doi: 10.1016/j.artmed.2012.12.006 (2013). 23395009 PMC4070079

[b35] Myte R. . Components of One-carbon Metabolism Other than Folate and Colorectal Cancer Risk. Epidemiology 27, 787–796, doi: 10.1097/EDE.0000000000000529 (2016). 27367522

[b36] Midttun O. . Most blood biomarkers related to vitamin status, one-carbon metabolism, and the kynurenine pathway show adequate preanalytical stability and within-person reproducibility to allow assessment of exposure or nutritional status in healthy women and cardiovascular patients. J Nutr 144, 784–790, doi: 10.3945/jn.113.189738 (2014). 24647388 PMC3985833

[b37] Hustad S. . Kinetic Modeling of Storage Effects on Biomarkers Related to B Vitamin Status and One-Carbon Metabolism. Clin Chem 58, 402–410, doi: 10.1373/clinchem.2011.174490 (2012). 22194632

[b38] Park J. Y. . Comparison of standardised dietary folate intake across ten countries participating in the European Prospective Investigation into Cancer and Nutrition. Br J Nutr 108, 552–569, doi: 10.1017/S0007114511005733 (2012). 22040523

[b39] Johansson I. . Validity of food frequency questionnaire estimated intakes of folate and other B vitamins in a region without folic acid fortification. Eur J Clin Nutr 64, 905–913, doi: 10.1038/ejcn.2010.80 (2010). 20502473

[b40] Eussen S. J. . North-south gradients in plasma concentrations of B-vitamins and other components of one-carbon metabolism in Western Europe: results from the European Prospective Investigation into Cancer and Nutrition (EPIC) Study. Br J Nutr 110, 363–374, doi: 10.1017/S0007114512004990 (2013). 23228223

[b41] Kalmbach R. D. . Circulating folic acid in plasma: relation to folic acid fortification. Am J Clin Nutr 88, 763–768 (2008). 18779294 10.1093/ajcn/88.3.763 PMC3763811

[b42] Van Guelpen B. . Plasma folate and total homocysteine levels are associated with the risk of myocardial infarction, independently of each other and of renal function. J Intern Med 266, 182–195, doi: 10.1111/j.1365-2796.2009.02077.x (2009). 19298497

[b43] Weinehall L., Hallgren C. G., Westman G., Janlert U. & Wall S. Reduction of selection bias in primary prevention of cardiovascular disease through involvement of primary health care. Scand J Prim Health Care 16, 171–176 (1998). 9800231 10.1080/028134398750003133

[b44] Pukkala E. . Nordic biological specimen banks as basis for studies of cancer causes and control–more than 2 million sample donors, 25 million person years and 100,000 prospective cancers. Acta Oncol 46, 286–307, doi: 10.1080/02841860701203545 (2007). 17450464

[b45] Eklof V. . The reduced folate carrier (RFC1) 80G > A and folate hydrolase 1 (FOLH1) 1561C > T polymorphisms and the risk of colorectal cancer: a nested case-referent study. Scand J Clin Lab Invest 68, 393–401, doi: 10.1080/00365510701805431 (2008). 19172696

[b46] Eussen S. J. . Plasma folate, related genetic variants, and colorectal cancer risk in EPIC. Cancer Epidemiol Biomarkers Prev 19, 1328–1340, doi: 10.1158/1055-9965.EPI-09-0841 (2010). 20447924 PMC2880712

[b47] BEVITAL. [*Internet*], http://www.bevital.no (2003–2004).

[b48] Midttun O., Hustad S. & Ueland P. M. Quantitative profiling of biomarkers related to B-vitamin status, tryptophan metabolism and inflammation in human plasma by liquid chromatography/tandem mass spectrometry. Rapid Commun Mass Spectrom 23, 1371–1379, doi: 10.1002/rcm.4013 (2009). 19337982

[b49] Windelberg A., Arseth O., Kvalheim G. & Ueland P. M. Automated assay for the determination of methylmalonic acid, total homocysteine, and related amino acids in human serum or plasma by means of methylchloroformate derivatization and gas chromatography-mass spectrometry. Clin Chem 51, 2103–2109, doi: 10.1373/clinchem.2005.053835 (2005). 16123148

[b50] Molloy A. M. & Scott J. M. Microbiological assay for serum, plasma, and red cell folate using cryopreserved, microtiter plate method. Methods Enzymol 281, 43–53 (1997). 9250965 10.1016/s0076-6879(97)81007-5

[b51] Kelleher B. P. & Broin S. D. Microbiological assay for vitamin B12 performed in 96-well microtitre plates. J Clin Pathol 44, 592–595 (1991). 1856292 10.1136/jcp.44.7.592 PMC496801

[b52] Meyer K., Fredriksen A. & Ueland P. M. MALDI-TOF MS genotyping of polymorphisms related to 1-carbon metabolism using common and mass-modified terminators. Clin Chem 55, 139–149, doi: 10.1373/clinchem.2008.115378 (2009). 18988749

[b53] Sucher R. . Neopterin, a prognostic marker in human malignancies. Cancer Lett 287, 13–22, doi: 10.1016/j.canlet.2009.05.008 (2010). 19500901

[b54] Chen Y. & Guillemin G. J. Kynurenine pathway metabolites in humans: disease and healthy States. Int J Tryptophan Res 2, 1–19 (2009). 22084578 10.4137/ijtr.s2097 PMC3195227

[b55] R Development Core Team. R: A Language and Environment for Statistical Computing (R Foundation for Statistical Computing, 2012).

[b56] Shannon P. . Cytoscape: A software environment for integrated models of biomolecular interaction networks. Genome Res 13, 2498–2504 (2003). 14597658 10.1101/gr.1239303 PMC403769

[b57] Wang M. . Statistical methods for studying disease subtype heterogeneity. Stat Med 35, 782–800 (2016). 26619806 10.1002/sim.6793 PMC4728021

