# Supplementary material for: Untangling the role of one-carbon metabolism in colorectal cancer risk: a comprehensive Bayesian network analysis
Source: Sci Rep. 2017 Feb 24;7:43434. doi: 10.1038/srep43434 (PMC5324061; doi:10.1038/srep43434)
Supplement: Supplementary Materials [file srep43434-s1.pdf]

Untangling the role of one-carbon metabolism in colorectal cancer risk: a comprehensive Bayesian network analysis

Robin Myte, Björn Gylling, Jenny Häggström, Jörn Schneede, Per Magne Ueland, Göran Hallmans, Ingegerd Johansson, Richard Palmqvist, Bethany Van Guelpen

SUPPLEMENTARY MATERIALS

Supplementary Table S1 Genotype distributions of single nucleotide polymorphisms related to one-carbon metabolism in colorectal cancer cases and controls and tests of Hardy-Weinberg equilibrium.

Supplementary Table S2 Strongest relations to CRC risk within subgroups.

Supplementary Figure S1 Bivariate correlations between log-transformed plasma concentrations of one-carbon metabolites calculated with Spearman's correlation coefficient.

Supplementary Table S1 Genotype distributions of single nucleotide polymorphisms related to one-carbon metabolism.

SNP		Genotype Common	Heterozygous	Homozygous	P_{HW}[a]	P[b]	Missing (%)
CBS 844ins68[c]	Cases	554 (91%)	54 (9%)	1 (0%)	1.000	0.51	1
	Controls	1045 (89%)	125 (11%)	3 (0%)	1.000		
CBS 699C>T	Cases	283 (47%)	257 (42%)	68 (11%)	0.447	0.94	1
	Controls	540 (46%)	504 (43%)	127 (11%)	0.598		
MTHFR 677C>T	Cases	326 (53%)	232 (38%)	53 (9%)	0.236	0.23	0.6
	Controls	580 (49%)	489 (41%)	112 (9%)	0.581		
MTHFR 1298 A>C	Cases	253 (41%)	275 (45%)	83 (14%)	0.594	0.27	0.6
	Controls	496 (42%)	556 (47%)	130 (11%)	0.179		
MTR 2756A>G	Cases	375 (62%)	192 (32%)	42 (7%)	0.017	0.30	1
	Controls	685 (58%)	413 (35%)	75 (6%)	0.260		
MTRR 66A>G	Cases	108 (18%)	288 (47%)	211 (35%)	0.615	0.32	1
	Controls	195 (17%)	600 (51%)	376 (32%)	0.097		
MTRR 524 C>T	Cases	250 (42%)	275 (46%)	77 (13%)	0.968	0.63	2
	Controls	505 (44%)	521 (45%)	134 (12%)	0.973		
BHMT 742 G>A	Cases	297 (49%)	264 (43%)	47 (8%)	0.300	0.89	2
	Controls	580 (50%)	494 (42%)	94 (8%)	0.465		
TCII 776 C>G	Cases	220 (36%)	295 (49%)	93 (15%)	0.766	0.54	1
	Controls	432 (37%)	540 (46%)	199 (17%)	0.189		
TCII 67 A>G	Cases	439 (72%)	161 (26%)	8 (1%)	0.147	0.31	1
	Controls	835 (71%)	308 (26%)	28 (2%)	0.967		
RFC1 80G>A	Cases	173 (29%)	268 (44%)	162 (27%)	0.008	0.09	2
	Controls	335 (29%)	571 (49%)	262 (22%)	0.558		
FOLR1 1413G>A[c]	Cases	559 (92%)	47 (8%)	0 (0%)	1.000	0.65	2
	Controls	1065 (92%)	94 (8%)	3 (0%)	0.468		
MTHFD1 1958G>A	Cases	156 (26%)	317 (52%)	136 (22%)	0.327	0.18	1
	Controls	347 (30%)	566 (48%)	260 (22%)	0.332		
CTH 1364G>T	Cases	366 (61%)	202 (34%)	34 (6%)	0.437	0.80	2
	Controls	688 (59%)	403 (35%)	71 (6%)	0.274		
SHMT1 1420C>T	Cases	285 (47%)	242 (40%)	77 (13%)	0.031	1.00	2
	Controls	553 (47%)	466 (40%)	148 (13%)	0.002		
DHFR 19 del	Cases	189 (32%)	291 (49%)	117 (20%)	0.841	0.15	3
	Controls	352 (31%)	525 (46%)	272 (24%)	0.006		
TYMS 6 del	Cases	272 (45%)	245 (41%)	83 (14%)	0.027	0.44	3
	Controls	511 (44%)	499 (43%)	139 (12%)	0.339		

[a] From Chi-square test for Hardy-Weinberg equilibrium (Bonferroni-corrected threshold for significance = 0.05/34 ≈ 0.0015

[a] From Chi-square test for difference in genotype distribution between cases and controls (Bonferroni-corrected threshold for significance = 0.05/17 ≈ 0.003).

[c] Expected cell counts in Chi-square test below 5, therefore P-values are from a Fisher's exact test.

Supplementary Table S2 Strongest relations to CRC risk within subgroups.

Subgroup	Cases/controls (n)	Top 3 variables (confidence)[a]			Threshold[b]
Males	253/487	Folate<br>54%	B2<br>33%	RFC1 80G>A<br>28%	50%
Females	360/703	B2<br>40%	MTHFD1 1958G>A<br>29%	DHFR 19 del<br>25%	51%
Follow-up <8.2 y	306/584	B6<br>33%	DHFR 19 del<br>30%	B2<br>20%	49%
Follow-up ≥8.2 y	307/606	Betaine<br>35%	Folate<br>31%	RFC1 80G>A<br>26%	50%
Right Colon	183/357	RFC1 80G>A<br>37%	Methionine<br>22%	DHFR 19 del<br>21%	53%
Left Colon	215/419	CBS 699C>T<br>41%	Folate<br>33%	MTRR 66A>G<br>25%	52%
Rectum	214/412	B12<br>51%	MTRR 66A>G<br>23%	MTHFR 677C>T<br>22%	51%
Stage I&II	308/600	DHFR 19 del<br>40%	MTRR 66A>G<br>27%	RFC1 80G>A<br>24%	49%
Stage III&IV	276/533	Folate<br>57%	B2<br>27%	Choline<br>25%	50%

[a] Edge confidences to CRC in subgroups (i.e. frequency of the relation in the 1000 bootstrap samples), for the top three most influential one-carbon metabolism variables in Bayesian networks estimated using the HC algorithm.
[b] Threshold for an edge to be included in the final network estimated based on the observed distribution of edge confidences

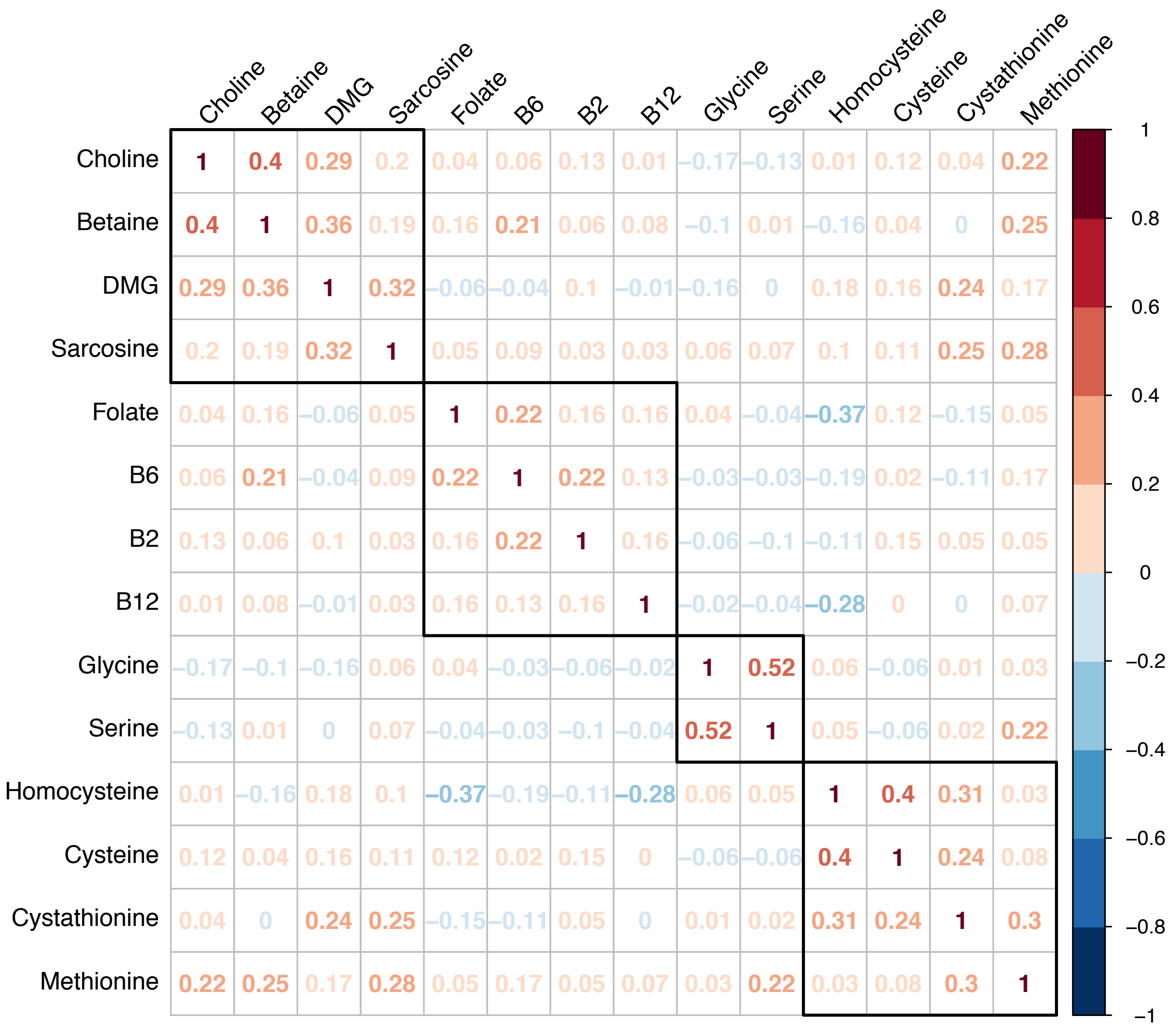

Supplementary Figure S1 Correlations between plasma one-carbon metabolites. Calculated with Spearman's correlation coefficient on pairwise complete observations. Rectangles represent four clusters from a hierarchical cluster analysis using Spearman's correlation as distance measure with complete linkage. Abbreviations: DMG, dimethylglycine - B6, vitamin B6 (PLP, pyridoxal 5' phosphate) - B2, vitamin B2 (riboflavin) - B12, vitamin B12 (cobalamin).
